# Prevalence and characteristics of needlestick injuries among dental interns during their first-year clinical training: an observational study

**DOI:** 10.1186/s12903-023-02892-5

**Published:** 2023-04-02

**Authors:** Jinwei Huang, Yena Gan, He Xu, Nan Li, Na An, Zhigang Cai

**Affiliations:** 1 Department of General Dentistry II, Peking University School and Hospital of Stomatology, Beijing, 100081 China; 2 National Center for Stomatology, Beijing, China; 3 National Clinical Research Center for Oral Diseases, Beijing, China; 4 National Engineering Research Center of Oral Biomaterials and Digital Medical Devices, Beijing, China; 5 Beijing Key Laboratory of Digital Stomatology, Beijing, China; 6 Research Center of Engineering and Technology for Computerized Dentistry Ministry of Health, Beijing, China; 7 Department of Tuina and Pain, Dongzhimen Hospital, Beijing University of Chinese Medicine, Beijing, China; 8 Department of Paediatric Dentistry, Peking University School and Hospital of Stomatology, Beijing, China; 9 Department of Epidemiology and Biostatistics, School of Public Health, Peking University Health Science Center, Beijing, China; 10 Department of Oral and Maxillofacial Surgery, Peking University School and Hospital of Stomatology, Beijing, 100081 China

**Keywords:** Needlestick injury, First-year, Dental intern, Occupation exposure, Infection control

## Abstract

**Background:**

Dental interns are vulnerable to needlestick injuries (NSI). The objectives of this study were to examine the prevalence and characteristics of NSI exposures among dental interns during their first-year clinical training, assess risk factors, and evaluate reporting behaviours.

**Methods:**

An online survey was conducted among dental interns of Class 2011–2017 at Peking University School and Hospital of Stomatology (PKUSS), China. The self-administrated questionnaire consisted of information on demographic profiles, NSI characteristics, and reporting practices. The outcomes were presented by descriptive statistics. A multivariate regression analysis was performed to assess NSI sources using a forward step-wise approach.

**Results:**

A total of 407 dental interns completed the survey (response rate 91.9%, 407/443), and 23.8% sustained at least one NSI. The mean number of NSIs per intern was 0.28 during the first clinical year. More occupation exposures occurred from October to December, between 13:00–15:00. Syringe needles were the most frequent sources, followed by dental burs, suture needles, and ultrasonic chips. The risk of peer-inflicted NSIs in the department of Paediatric Dentistry was 12.1 times higher than that in Oral Surgery (OR 12.1, 95% CI: 1.4-101.4). Appropriately 64.9% NSIs occurred when chairside assistants were absent. Compared to working alone, the risk of peer-inflicted NSIs was 32.3 times higher when providing chairside assistance (OR 32.3, 95% CI: 7.2-145.4). The left-hand index finger was the most commonly injured site. About 71.4% of exposures were reported in paperwork.

**Conclusions:**

Dental interns are susceptible to NSIs during their first-year clinical training. Extra attention should be paid to syringe needles, dental burs, suture needles, and ultrasonic chips. The lack of chairside assistance is hazardous regarding NSIs. The training of chairside assistance of the first-year dental interns should be enhanced. First-year dental interns are required to increase their awareness of ignored behaviors related to NSI exposures.

## Background

The oral cavity is a fertile environment for the transmission, inoculation, and growth of various infectious agents, including pathogenic bloodborne viruses such as hepatitis B virus (HBV), human immunodeficiency virus (HIV), and severe acute respiratory syndrome coronavirus 2 (SARS-CoV-2). The limited visualization and access, frequent use of aerosol-generated instruments, and close contact between dentist and patient during treatment procedures, contribute to the increased vulnerability of dental professionals to infectious diseases [1, 2]. The worldwide HBV infection rate was higher in dentists than in the general population: 6 times higher in the USA, 4 times higher in Germany, and 2.5 times higher in Japan [3]. Multiple transmission routes of pathogenic microorganisms have been identified during the delivery of dental care: (1) direct contact with blood, oral fluids, or other patient materials; (2) indirect contact with contaminated objects (e.g., instruments, equipment, surfaces); (3) contact of conjunctival, nasal, or oral mucosa with droplets from an infected person and propelled by coughing, sneezing, talking or using dental instruments; as well as (4) inhalation of airborne microorganisms in the environment [4].

Through the direct or indirect contact transmission route, needlestick injuries (NSI) were one of the main causes of the pathogenic transmission of viruses. A needlestick injury was defined as any contact of non-intact skin, eye, mucous membrane, or parenteral contact (e.g., needlestick, cut, abrasion, instrument puncture) with blood or any other potentially infectious material (e.g., saliva) [5–7]. NSIs pose a definite risk of bloodborne virus infection for all dental care providers, especially among dental interns who have less experience in infection control procedures and are frequently obliged to work on patients without assistance [6]. Depending on the source of the NSIs, the occurrence of exposures might be self-inflicted or caused by others. In a university hospital in Taiwan, more than two-thirds of NSI cases were self-inflicted, while about 30% were induced by other personnel or patients [8]. Numerous published studies have reached a consensus that dental interns are at a high risk of NSI exposure and necessary interventions are advocated to decrease the prevalence [9–13]. However, even though an alarming frequency was observed, the distribution of NSIs among dental interns of different academic years seemed to be varied. In particular, junior interns sustained more exposure compared to senior interns [7].

To the best of our knowledge, former studies focused mainly on investigating NSI prevalence among the whole dental student community, from pre-clinical to clinical training. Our previous studies indicated that NSI exposures among first-year dental interns demonstrated unique characteristics compared with those in other grades [13, 14]. The precise risk factors of NSIs in this group were investigated less frequently. Thus, to provide necessary information for freshman dental interns, we examined the prevalence and characteristics of self-reported NSI exposures among dental interns during their first-year clinical training, assessed risk factors, and evaluated reporting behaviours.

## Methods

### Study design and participants

A cross-sectional survey based on a self-administrated questionnaire was conducted among dental interns of Peking University School and Hospital of Stomatology (PKUSS), which is a major tertiary academic teaching hospital located in Beijing, China. The classes of 2011–2017 were invited to participate in this study. For classes of 2015–2017, the survey was performed at the end of their first-year clinical training, whilst for the classes of 2011–2014, the information on NSI exposures was derived retrospectively in June 2020. All participants engaged in full-time general dentistry training under the supervision of senior faculty members in their fifth year of education, which was the first year of their clinical training. The training included basic concepts and procedures in Cariology and Endodontology, Periodontology, Paediatric Dentistry, Prosthodontics, and Oral Surgery. The study was performed following the Strengthening the Reporting of Observational Studies in Epidemiology (STROBE) guidelines [15] and in accordance with the Declaration of Helsinki. It had been approved by the Ethics Committee of PKUSS (PKUSSIRB-202056081).

### Survey instrument

A self-administrated questionnaire was developed and modified according to published studies, which had been used in our previous study [13]. The questionnaire was highly structured and consisted of four domains: demographic profile (e.g., sex, age, seniority); NSI characteristics (e.g., causative instrument and procedure involved, anatomic site of injury, presence of assistant); psychological reaction after exposures; and reporting practices. The definition of NSI was included at the beginning of the questionnaire for clarity in answering the questions. Participation was entirely voluntary and completely anonymous, thereby guaranteeing the confidentiality of the data obtained. Informed consent was included on the front page of the questionnaire, and completion of the questionnaire implied providing consent for study participation.

### Statistical analysis

Survey responses were coded and counted in descriptive statistic form to describe the characteristics of the study population and the NSI exposures. The count data were summarized as frequencies and percentages. The quantitative data were presented as means ± standard deviations (min, max), whereas parameters with non-Gaussian distribution were expressed as medians and interquartile ranges (Q1–Q3) as appropriate. The associations of sex and class with NSI exposures were estimated with a relative risk (RR) and 95% confidence intervals (CI). Differences were considered significant when the 95% CI did not contain 1 for RR. The chi-squared and Fisher exact tests were used for categorical variables, while the Wilcoxon rank sum test for continuous variables. NSI sources (self-inflicted VS. peer-inflicted) were assessed by logistic regression. A multivariate regression analysis was performed for significant variables in the univariate analysis using a forward step-wise approach. The results were presented as an odd ratio (OR) with 95% CI. P-value < 0.05 was considered statistically significant. All data were analysed using R software version 4.2.0 (R Foundation for Statistical Computing, Vienna, Austria), and subsequently imported into Microsoft Visio Pro version 2019 (Microsoft Corporation, Washington, USA) for the creation of appropriate graphs.

## Results

### Demographics of participants and NSI exposure rates

A total of 443 dental interns were invited to participate in the survey and 407 completed the questionnaire, resulting in an overall response rate of 91.9%. The study cohort comprised 153 (37.6%) male and 254 (62.4%) female interns, aged 21–26 years when they entered their first year of clinical training. Out of the 407 respondents, 97 (23.8%) sustained at least one NSI exposure, including 32 male and 65 female interns. Among these injured interns, 81 of them reported one single accident, 15 stated two times of exposure, and 1 suffered three times. A total of 114 NSI exposures were reported, thus the mean number of NSIs per intern was 0.28 (114/407) during the first year of clinical training.

### Potential associated factors of NSI exposures

No statistical difference was found between male and female interns regarding NSI exposures (RR = 0.83, 95%CI: 0.58–1.18). Taking Class 2011 as the baseline reference, the risk of NSI exposures significantly increased: 0.73 times for Class 2012 (RR = 1.73, 95%CI: 1.01–2.97) and 2014 (RR = 1.73, 95%CI: 1.09–2.75), 0.60 times for Class 2016 (RR = 1.60, 95%CI: 1.02–2.51), and 0.72 times for Class 2017 (RR = 1.72, 95%CI: 1.25–2.37). Although no statistical difference was observed, the risk of NSI exposures also increased by 0.31 and 0.59 times for Classes 2013 and 2015, respectively (Table 1).

**Table 1:** Characteristics of the participating dental interns and NSI prevalence during their first-year clinical training by sex and class

Class	Sex	Response rate n (%)	Respondents with NSIs n (%)	Total number of NSIs (n=)	RR (95%CI) (exposed vs. non-exposed)	
					Sex	Class
2011	Male	21 (100.0)	2 (9.5)	2	1.14 (0.18, 7.46)	As baseline
	Female	36 (87.8)	3 (8.3)	4		
2012	Male	19 (79.2)	2 (10.5)	2	0.35 (0.09, 1.34)	1.73 (1.01, 2.97)
	Female	29 (78.4)	10 (34.5)	10		
2013	Male	19 (95.0)	4 (21.1)	4	1.60 (0.39, 6.48)	1.31 (0.53, 3.23)
	Female	30 (85.7)	3 (10.0)	6		
2014	Male	26 (86.7)	8 (30.8)	9	1.19 (0.30, 4.70)	1.73 (1.09, 2.75)
	Female	30 (96.8)	8 (26.7)	10		
2015	Male	19 (100.0)	3 (15.8)	3	0.38 (0.09, 1.61)	1.59 (0.99, 2.55)
	Female	40 (100.0)	12 (30.0)	13		
2016	Male	22 (100.0)	6 (27.3)	6	1.18 (0.36, 3.92)	1.60 (1.02, 2.51)
	Female	39 (100.0)	9 (23.1)	13		
2017	Male	27 (87.1)	7 (25.9)	10	0.65 (0.28, 1.53)	1.72 (1.25, 2.37)
	Female	50 (94.3)	20 (40.0)	22		
Sum	Male	153 (91.6)	32 (20.9)	36	0.83 (0.58, 1.18)	—
	Female	254 (92.0)	65 (25.6)	78		

NSI, needlestick injury; RR, relative risk

**Fig. 1:**
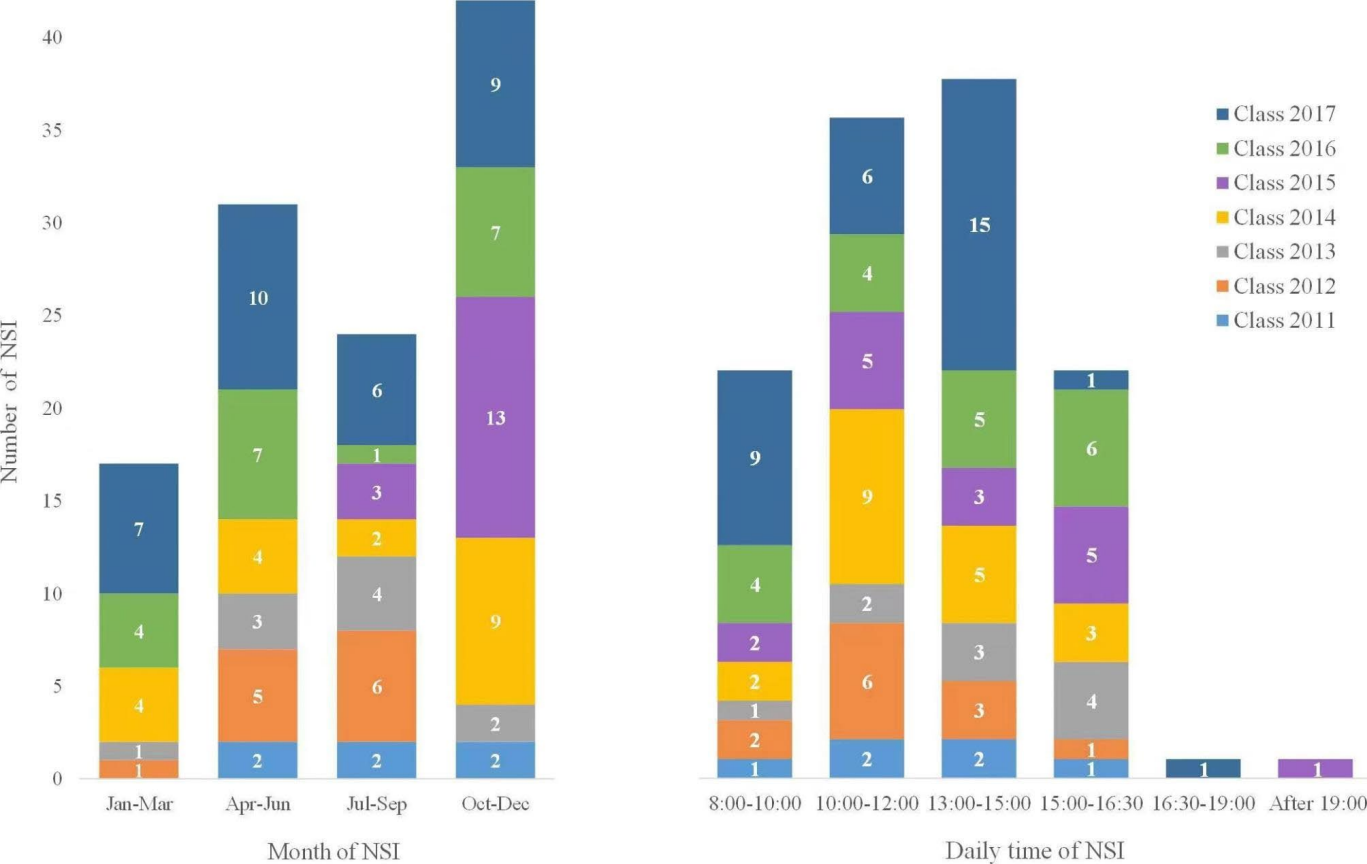
Timing distribution of NSI exposures among dental interns during their first-year clinical training. NSI, needlestick injury

At the time of exposure, the mean age of the injured interns was 22.6 ± 0.9 (21, 26) years, and they were working for 6.3 ± 1.9 (2, 13) hours per day. Up to 93.3% of the dental interns were right-handed, and there was no statistical difference between right- and left-handed interns regarding NSI events (p = 0.2593). More exposures were observed from October to December. In daily time, the majority of NSI exposures occurred between 13:00–15:00 and 10:00–12:00 (Fig. 1).

When recapping the syringe needles, 91.2% of the injured interns used the one-handed recapping technique. However, the vast majority of incidents were reported to be associated with syringe needles (26.3%), followed by dental burs (22.8%), suture needles (14.0%), and ultrasonic chips (13.2%). Periodontal treatment was documented as the most frequent cause of NSI exposures (18.4%), while no exposure related to prosthodontology was reported.

### Peer-inflicted NSIs and its risk factors

The source of the NSI could be divided into self-inflicted and peer-inflicted.Univariate and multivariate analysis revealed that the specialties and chairside assistance were statistically significantly associated with the peer-inflicted NSIs (Tables 2 and 3). The risk of peer-inflicted NSIs in the department of Paediatric Dentistry was 12.1 times higher than that in the department of Oral Surgery (OR 12.1, 95% CI: 1.4-101.4) (Table 3). Up to 76.2% of the peer-inflicted NSIs occurred when providing chairside assistance (OR 32.3, 95% CI: 7.2-145.4) (Table 3), indicating that the training of chairside assistance might be inadequate for the first-year dental interns.

**Table 2:** Univariate analysis of factors associated with peer-inflicted NSIs among first-year dental interns

Variables	Self-inflicted (n = 93)	Peer-inflicted (n = 21)	p-value OR (95% CI)
**Sex**			
Male	32 (34.4%)	4 (19.0%)	As baseline
Female	61 (65.6%)	17 (81.0%)	0.179 2.2 (0.7, 7.2)
**Dominant hand**			
Right-handed	88 (94.6%)	18 (85.7%)	As baseline
Left-handed	5 (5.4%)	3 (14.3%)	0.165 2.9 (0.6, 13.4)
**Month**			
January-March	14 (15.1%)	3 (14.3%)	As baseline
April-June	25 (26.9%)	6 (28.6%)	0.885 1.1 (0.2, 5.2)
July-September	21 (22.6%)	3 (14.3%)	0.647 0.7 (0.1, 3.8)
October-December	33 (35.5%)	9 (42.9%)	0.744 1.3 (0.3, 5.4)
**Daily time**			
Morning	45 (48.4%)	10 (47.6%)	As baseline
Afternoon	48 (51.6%)	11 (52.4%)	0.949 1.0 (0.4, 2.7)
**Specialties**			
Oral Surgery	32 (34.4%)	3 (14.3%)	As baseline
Cariology and Endodontology	26 (28.0%)	5 (23.8%)	0.334 2.1 (0.5, 9.7)
Periodontology	27 (29.0%)	5 (23.8%)	0.359 2.0 (0.4, 9.3)
Paediatric Dentistry	7 (7.5%)	6 (28.6%)	**0.004** ^*^ 11.0 (2.1, 56.5)
**Role**			
Dentist	75 (80.6%)	17 (81.0%)	As baseline
Assistant	18 (19.4%)	4 (19.0%)	0.974 1.0 (0.3, 3.3)
**Anatomic sites of NSI**			
Forearm	3 (3.2%)	1 (4.8%)	As baseline
Thumb	21 (22.6%)	0 (0.0%)	0.990 0.0 (0.0, Inf)
Index finger	33 (35.5%)	7 (33.3%)	0.713 0.6 (0.1, 7.1)
Middle finger	9 (9.7%)	3 (14.3%)	1.000 1.0 (0.1, 13.6)
Ring finger	2 (2.2%)	3 (14.3%)	0.307 4.5 (0.3, 80.6)
Palm	6 (6.5%)	3 (14.3%)	0.765 1.5 (0.1, 21.3)
Hand dorsum	14 (15.1%)	3 (14.3%)	0.738 0.6 (0.0, 8.5)
**Body side**			
Right	50 (53.8%)	15 (71.4%)	As baseline
Left	43 (46.2%)	6 (28.6%)	0.146 0.5 (0.2, 1.3)
**Instruments**			
Syringe needle	23 (24.7%)	7 (33.3%)	As baseline
Suture needle	16 (17.2%)	0 (0.0%)	0.991 0.0 (0.0, Inf)
Ultrasonic chip	11 (11.8%)	4 (19.0%)	0.806 1.2 (0.3, 5.0)
Surgical scalpel	2 (2.2%)	3 (14.3%)	0.114 4.9 (0.7, 35.7)
Periodontal scaler	10 (10.8%)	0 (0.0%)	0.993 0.0 (0.0, Inf)
Dental bur	22 (23.7%)	4 (19.0%)	0.458 0.6 (0.2, 2.3)
Endodontic file	4 (4.3%)	1 (4.8%)	0.870 0.8 (0.1, 8.6)
Others	5 (5.4%)	2 (9.5%)	0.772 1.3 (0.2, 8.3)
**Procedures**			
Local anesthesia	9 (9.7%)	3 (14.3%)	As baseline
Surgical operation	23 (24.7%)	3 (14.3%)	0.301 0.4 (0.1, 2.3)
Endodontic treatment	19 (20.4%)	4 (19.0%)	0.595 0.6 (0.1, 3.4)
Periodontal treatment	17 (18.3%)	4 (19.0%)	0.688 0.7 (0.1, 3.9)
Chairside assistance	19 (20.4%)	5 (23.8%)	0.777 0.8 (0.2, 4.1)
Others	6 (6.5%)	2 (9.5%)	1.000 1.0 (0.1, 7.9)
**Perioperative staging**			
Pre-procedures	10 (10.9%)	1 (5.0%)	As baseline
During procedures	44 (47.8%)	7 (35.0%)	0.680 1.6 (0.2, 14.4)
Post-procedures	38 (41.3%)	12 (60.0%)	0.296 3.2 (0.4, 27.3)
**Assistance**			
Working alone	70 (76.1%)	4 (19.0%)	As baseline
Working with assistance	10 (10.9%)	1 (4.8%)	0.632 1.7 (0.2, 17.3)
Providing assistance	12 (13.0%)	16 (76.2%)	**< 0.001** ^*^ 23.3 (6.7, 81.9)
**Recapping of needles**			
One-handed	84 (90.3%)	20 (95.2%)	As baseline
Two-handed	9 (9.7%)	1 (4.8%)	0.482 0.5 (0.1, 3.9)
**Daily number of patients/day**	3.0 (2,4)	2.5 (2,4)	0.298 0.8 (0.5, 1.3)
**Daily working time/h**	6.2 ± 1.8 (2,12)	6.8 ± 2.4 (2,13)	0.203 1.2 (0.9, 1.5)

NSI, needlestick injury; ^*^p-value<0.05

**Table 3:** Multivariate logistic regression for risk factors identified in the univariate analysis

Variables	Adjusted OR (95% CI)	p-value
**Specialties**		
Oral Surgery	As baseline	
Cariology and Endodontology	3.9 (0.6, 25.3)	0.151
Periodontology	2.2 (0.4, 13.2)	0.373
Pediatric Dentistry	12.1 (1.4, 101.4)	**0.022** ^*^
**Assistance**		
Working alone	As baseline	
Working with assistance	2.5 (0.2, 28.9)	0.459
Providing assistance	32.3 (7.2, 145.4)	**< 0.001** ^*^

^*^p-value<0.05.

### Anatomic sites of NSIs

**Fig. 2:**
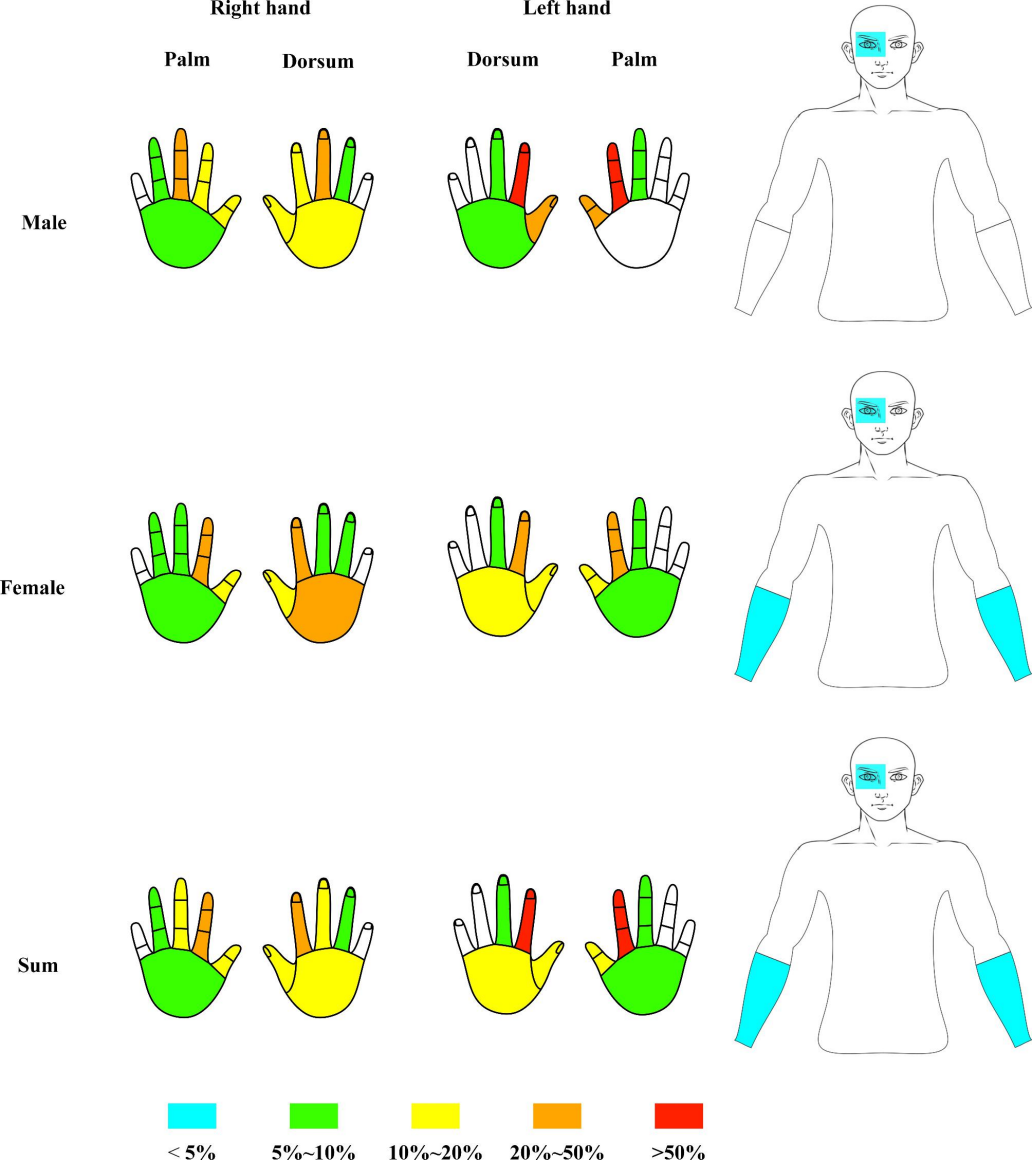
The anatomic sites of NSI exposures of injured dental interns during the first-year clinic training. NSI, needlestick injury

The injured anatomic sites were categorized into the thumb, index finger, middle finger, ring finger, little finger, palm, hand dorsum, forearm, eye, etc. Different colours represented different proportions of NSIs, while blank space indicated no cases were reported. The most vulnerable site of NSI was the left-hand index finger, observed in over 50% and 20% of male and female interns, respectively. The right-hand index finger was also susceptible to NSIs, which covered 20–50% of the overall exposures (Fig. 2).

### Perceived causes of NSI, post-exposure management, and psychological reaction

Around one-fourth of dental interns in this cohort experienced NSI events, and the most common cause contributors were lapse in concentration (57.9%), followed by lack of time (19.3%), lack of technical training or supervision (19.3%), and fatigue (14.0%) (Table 4). Notably, 71.4% (80/114) of the exposures were reported to the designated faculty member. Among these 80 reported cases, blood tests were performed for 79 cases; however, only 4 cases required medical interventions. For those under-reporting cases, the most commonly cited reasons were “the exposure was not significant” (32.4%) or “the patient showed a low risk” (29.4%). After exposures, dental interns frequently felt anxious (49.1%), pressured (31.6%), fearful (28.9%), and had a sense of self-reproach (26.3%). However, 20.2% of them did “not care”.

**Table 4:** Causes of NSI exposures of dental interns during their first-year clinical training

Perceived causes of NSI	
Lapse in concentration	66 (57.9)
Lack of time	22 (19.3)
Lack of technical training or supervision	22 (19.3)
Fatigue	16 (14.0)
Injured by others	11 (9.6)
Poor cooperation of patients	5 (4.4)
Inexperience	5 (4.4)
Others	2 (1.8)

NSI, needlestick injury

## Discussion

Given the nature of the specialty, both dental professionals and the public believe that dental procedures are extremely hazardous in terms of infection transmission, especially during NSI events [16]. NSIs may result in substantial health consequences and psychological discomfort [17]. Healthcare workers with NSIs exhibited significantly higher levels of anxiety and depression than those who were unexposed [18]. Additionally, post-traumatic stress disorders were observed among those exposed care providers [19]. Long-term follow-up with serological tests and even medical intervention would inevitably impose an economic burden on the healthcare system.

The ‘profile and competences for the graduating European dentist’ document stated that dental graduates must have knowledge of cross-infection control and be competent at implementing cross-infection control in their practice [20]. However, even though standard precautions might be implemented, previous studies showed that dental interns had a higher incidence of NSIs compared to registered dentists or faculties [8, 21]. Dental students had nearly twice the number of NSIs compared with dentists with approximately ten-year working experience [1]. More than one-fifth of the interns sustained at least one NSI in their 1-year period of clinical training [8]. The distribution of occupation exposures seemed to be varied between different stages, and was more commonly observed among clinical interns than preclinical dental students [9]. More specifically, a higher proportion of accidents was noticed among dental interns in the early phases of clinical education [22, 23]. Thus, NSI events tend to occur in clinic interns with lesser skills and experience, in the period when they join the clinic as freshmen [8].

### Risk factors

Multiple factors have been reported to contribute to NSI exposures among dental interns, which include age, sex, year of admission, dominant hand, course specialty, lack of training, presence of chair-side assistance, fatigue, anxiety, and stress [9, 10, 13, 24]. The outcomes of this study indicated that no statistically significant association was found between NSIs and sex, age, or dominant hand, which was consistent with previous studies [7, 10, 24]. Taking Class 2011 as the baseline reference, the risk of NSI was rising for the Classes 2012, 2014, 2016, and 2017 in this survey. However, no statistical difference was observed for Classes 2013 and 2015. NSI was more frequently reported by female interns; it remains unclear whether female interns experienced a greater number of NSI exposures or whether they were more inclined to report NSI (or both) [10].

Various clinical procedures were involved with NSI exposures, including local anaesthesia, tooth cleaning or periodontal scaling, endodontic treatment, restorative treatment, surgical suture, waste disposal, surgical exodontia, chairside assistance, prosthodontic treatment, and paediatric dental treatment. The instruments of NSIs could be needles, dental burs, scalpels, perio scalers, endodontic files, surgical elevators, curettes, dental explorers, and orthodontic wires [13]. From a 19-year report of dental care providers in Japan, syringe needles were the most frequent cause, followed by suture needles, and ultrasonic scaler chips [25]. Not surprisingly, syringes and suture needles have also been documented as the most frequent cause of NSI among dental interns [7]. Specifically, the vast majority of exposures were inflicted by syringe needles both during surgical procedures and cleanup [8]. Therefore, more attention should be paid to the proper methods of handling or cleaning up sharp instruments [21]. Oral surgery clinics seemed to be a major source of exposure compared with other specialties, which could be explained by the invasive nature of the procedures owing to the use of sharp instruments. However, when considering the source of NSIs, the risk of peer-inflicted NSIs in the department of Paediatric Dentistry was even higher than that in the department of Oral Surgery (OR 12.1, 95% CI: 1.4-101.4). This outcome suggested that extra attention should be paid to the peer interns when practicing in the department of Paediatric Dentistry. Interestingly, no exposure related to prosthodontology was reported in this survey, which might be because of the lack of prosthodontic treatments in the teaching clinic as a result of the intrinsic self-paid nature. Oftentimes, most NSIs resulted from two-handed needle recapping when disposing of needles and syringes [24]. However, in this study, although 91.2% of the injured interns used the one-handed recapping technique, an exposure rate of 23.8% was observed. This might indicate that multiple factors combined contribute to the occurrence of NSI. In addition, dental burs could be another source of NSIs [9, 12], as well as perio scaling and polishing [24].

Insufficient clinical skills and experience were cited as the main risk factors for NSI [8, 13]. This conclusion is reinforced by the fact that the majority of multiple episodes of exposure occurred among interns who were in the initial phases of the clinical curriculum [10]. A two-fold incidence of NSI from July to September was observed since new interns begin their practice in that period [8]. In this survey, an obvious increase in NSI exposures was observed from October to December, which might be a result of increasing treatments provided after the transition period from preclinic to clinic training. Experience, dexterity, and skill contribute to reducing the risk of accidents resulting from unpredictable patient movements generated by physical or emotional discomfort during treatment [11]. Therefore this initial period requires extra attention on the prevention of NSI.

Another risk factor is the absence of appropriate chair-side assistance. Dental interns were frequently obliged to perform clinical procedures alone, which might increase the chance of NSI exposure [8]. Up to 76.5% of exposures occurred when the students were working unassisted [24], and statistical significance was observed [7]. In this survey, up to 64.9% of the NSIs occurred when there were no chairside assistants. All these data supported that the absence of chairside assistance was a definite risk factor for NSI occurrence. Besides, in this study, 76.2% of the peer-inflicted NSIs occurred when providing chairside assistance (OR 32.3, 95% CI: 7.2-145.4), which indicated that the training of chairside assistance was inadequate.

“Lapse in concentration” was cited as the most common contributor to NSI exposures for first-year dental interns, which accounted for 57.9% in this survey. However, in a study of medical and dental house officers, only 14.8% of exposures occurred because of “lapse in concentration”[26]. The first-year dental interns seemed less focused when practising, which might lead to an increasing of NSI exposures. Therefore, more specific training and remind are needed to improve their perceived self-efficacy and to reduce lapses in concentration.

### Reporting behaviours

Dental interns frequently lack the awareness that exposure incidents should be reported to the designated faculty. Over 90% of them claimed being unaware of any guideline or protocol for post-exposure management [24]. In a survey of 171 dental students, 56 (34%) participants experienced at least one occupational exposure, but only one was reported [12]. Underreporting was evident in approximately one-third of the first-year dental interns of the three-year programme [27]. Out of 167 individuals with exposures, 71.9% failed to report the accidents [11]. In this study, 71.4% of NSI exposures were reported, which was attributed to the continuous emphasis on adherence to post-exposure prophylaxis protocols. Additionally, the knowledge gap of infection control may lead to delays in appropriate post-exposure management [9]. In a meta-analysis on percutaneous injuries of dentists, developed countries reported more exposures than developing countries [28]. It might reflect the fact that better education in dental schools might lead to an increased reporting rate of occupational injuries when dental interns became registered dentists. Therefore, the high under-reporting rate argues for a re-evaluation of current policy. Student education should be reinforced to ensure that safe practices are observed, as well as stressing the importance of NSI reporting [29].

### Preventive strategy

The COVID-19 pandemic has made prevention of the SARS-CoV-2 the top priority in dentistry. The education on infection control has consequently been strengthened among dental educational institutions. During this crucial stage of their careers, it is advisable that dental interns be trained in developing effective prevention behaviour and habits, which would extend to their future practices. The traditional teaching of infection control to dental interns involves lectures and clinical training where the concepts and practical aspects are discussed and demonstrated by senior dental staff. Owing to the ever-changing evidence base, the content of the training packages should be updated based on published literature. Dental educational institutions therefore should be ready to modify their delivery and incorporate extra teaching to specifically address emerging areas [4].

According to the outcomes of this study, instruction on NSI prevention should be initiated early in clinical training. First-year dental interns need to pay more attention to ignored and potential risk behaviours. Regular education on the risks and safety precautions in clinical practice should be strictly maintained, especially in the dental curriculum and pre-occupational training course. Participation in case analysis of NSI whenever it occurs may help interns increase their knowledge and awareness in the prevention of NSI [8]. Periodic practical and verbal exams on personnel knowledge, attitude, and performance are also recommended.

A standard and easily accessible protocol should be formulated for post-exposure management. Exposure Prevention Information Network (EPINet™) was considered a valuable tool for monitoring NSIs, which provided uniform report forms [25]. It is suggested that dental interns analyse their own experiences of NSI from the perspective of infection control [10].

Since one-third of NSI exposures are needle-related, the establishment of a standard operating procedure for injection needle removal is necessary [8]. The one-handed recapping technique, removing the needle cap by using the thumb and index fingers, was recommended which could avoid the risk of rebound [14]. Moreover, it is suggested that the hub of the needle should be grabbed using a needle-holder or haemostat instead of with the fingers during needle disassembling [8]. A non-recapping policy with immediate disposal of either the conventional or safe syringe systems after injection was considered effective [30]. Evidence suggests that bur punctures were another main source of NSI when picking up an instrument from across the bracket table. Therefore, the placement of hand-piece holders and the bracket table should be adjusted. Additionally, dental students should form a habit of removing burs in a timely fashion [12].

More chairside assistance would be beneficial in reducing NSI events; however, since the employment of more assistants might not be possible, interns can be paired up for mutual aid and learning [8]. More importantly, it is mandatory to ensure that dental interns are introduced to the subject matter and assessed before they enter the clinical setting [12].

### Strengths and limitations

This study solely assessed dental interns during their first year of training; previous studies have indicated that they have more injuries than senior interns or attending dentists. Since most NSI exposures are preventable, detailed information about the circumstances of injuries is crucial in developing preventive interventions, especially for this special duration of training. Thus, the outcomes of this study are a cautionary note to remind dental interns who are just starting to treat patients on the risk of NSIs, and therefore be more attentive while performing their dental work. The findings provide a provisional report indicating the risky procedures and instruments that should be paid extra attention to, which represents the strength of this study.

However, the findings of this study should be interpreted with caution. The limitation of the study is the possibility of misclassification and recall bias of the survey-based methodology. The anonymous nature of the survey is expected to facilitate accurate reporting, and the exceptionally high response rate of the targeted group of interns might mitigate the biases. Another inherent limitation is the institutional bias based on the one-centre survey which might be more specific to our institution than to a larger population. Moreover, the regression analysis was performed based on data from the exposed group because of missing information from the unexposed group. Thus the outcomes of the statistical analysis were not sufficiently convincing for the whole sample of the study population.

## Conclusions

Dental interns are susceptible to NSI exposures, especially during their first-year clinical training. Syringe needles, dental burs, suture needles, and ultrasonic chips are the most notorious instruments and warrant extra attention when they are used during treatments. The index finger is the most vulnerable site of NSI, especially on the left hand. The lack of chairside assistance is crucial in the occurrence of NSI events. The training of chairside assistance of the first-year dental interns should be enhanced. The first-year dental interns are required to increase their awareness of ignored and potential risk behaviors related to NSI exposures. Dental educational institutions should incorporate additional teaching to the areas specifically addressed here. Regular education on safety precautions and monitoring of NSI events are highly suggested, especially in the dental curriculum and pre-occupational training course. The training in NSI prevention should be initiated at the earliest opportunity.

## Data Availability

All data generated or analysed during the current study are available from the corresponding authors on reasonable request.

## Abbreviations

NSI: needlestick injury
PKUSS: Peking University School and Hospital of Stomatology
STROBE: Strengthening the Reporting of Observational Studies in Epidemiology
HBV: hepatitis B virus
HIV: human immunodeficiency virus
SARS-CoV-2: severe acute respiratory syndrome coronavirus
SD: standard deviations
RR: relative risk
CI: confidence intervals
EPINet™: Exposure Prevention Information Network.
